# Managing ongoing swallow safety through information‐sharing: An ethnography of speech and language therapists and nurses at work on stroke units

**DOI:** 10.1111/1460-6984.12725

**Published:** 2022-04-09

**Authors:** Rachel Barnard, Julia Jones, Madeline Cruice

**Affiliations:** ^1^ School of Health Sciences, Division of Language and Communication Science City, University of London UK; ^2^ Centre for Research in Public Health and Community Care (CRIPACC), School of Health and Social Work University of Hertfordshire Hatfield UK; ^3^ School of Health Sciences, Division of Language and Communication Science City, University of London UK

**Keywords:** ethnography, interprofessional, nurses, safety, Speech and language therapists, stroke, swallowing

## Abstract

**Background:**

Speech and language therapists and nurses need to work together to keep patients with swallowing difficulties safe throughout their acute stroke admission. Speech and language therapists make recommendations for safe swallowing following assessment and nurses put recommendations into practice and monitor how patients cope. There has been little research into the everyday realities of ongoing swallow safety management by these two disciplines. Patient safety research in other fields of healthcare indicates that safety can be enhanced through understanding the cultural context in which risk decisions are made.

**Aims:**

To generate new understanding for how speech and language therapists (SLTs) and nurses share information for ongoing management of swallows safety on stroke units.

**Methods & Procedures:**

An ethnographic methodology involving 40 weeks of fieldwork on three stroke wards in England between 2015 and 2017. Fieldwork observation (357 h) and interviews with 43 members of SLT and nursing staff. Observational and interview data were analysed iteratively using techniques from the constant comparative method to create a thematically organized explanation.

**Outcomes & Results:**

An explanation for how disciplinary differences in time and space influenced how SLT and nursing staff shared information for ongoing management of swallow safety, based around three themes: (1) SLTs and nurses were aligned in concern for swallow safety across all information‐sharing routes; however, (2) ambiguity was introduced by the need for the information contained in swallowing recommendations to travel across time, creating dilemmas for nurses. Patients could improve or deteriorate after recommendations were made and nurses had competing demands on their time. Ambiguity had consequences for (3) critical incident reporting and relationships. SLTs experienced dilemmas over how to act when recommendations were not followed.

**Conclusions & Implications:**

This study provides new understanding for patient safety dilemmas associated with the enactment and oversight of swallowing recommendations in context, on stroke wards. Findings can support SLTs and nurses to explore together how information for ongoing dysphagia management can be safely implemented within ward realities and kept up to date. This could include considering nursing capacity to act when SLTs are not there, mealtime staffing and SLT 7‐day working. Together they can review their understanding of risk and preferred local and formal routes for learning from it.

**What this paper adds:**

**What is already known on the subject**

- It is known that information to keep swallowing safe is shared through swallowing recommendations, which are understood to involve a balance of risks between optimizing the safety of the swallow mechanism and maintaining physiological and emotional health. There is increasing appreciation from patient safety research, of the importance of understanding the context in which hospital staff make decisions about risk and patient safety.

**What this paper adds to existing knowledge**

- The paper provides new empirical understanding for the complexities of risk management associated with SLT and nursing interactions and roles with respect to ongoing swallow safety.

**What are the potential or actual clinical implications of this work?**

- Findings can underpin SLT and nurse discussion about how swallow safety could be improved in their own settings.

## INTRODUCTION

### Background

Oro‐pharyngeal dysphagia is extremely common at the onset of stroke and is associated with pneumonia, increased mortality, longer hospital stays and increased disability on discharge from hospital (Al‐Khaled et al., 2016). In many countries management of dysphagia is led by speech and language therapists (SLTs), who conduct comprehensive assessment and provide advice for patients to reduce the risk of aspiration or choking. SLTs work closely with nurses, who perform essential roles in identifying signs of dysphagia through screening and ensuring safe swallowing recommendations are followed, with consideration for their effect on patients’ wider health and emotional needs (Atkinson & O'Kane, 2018). Recommendations might include modifying how food and drink are delivered (e.g., slower pace), attention to positioning, and modifying food and fluid textures and consistencies, such as adding thickening agents to allow more time for airway closure by slowing the transit of fluids (Steele et al., 2015).

Dysphagia management needs to operate through balancing risks. There is growing appreciation that dysphagia interventions may in themselves cause harm, particularly when overly focused on textural modifications with lesser consideration of the wider health picture (O'Keefe, 2018). For example, thickening fluids can negatively affect hydration, nutrition, medication absorption and quality of life (Atkinson & O'Kane, 2018; O'Keefe, 2018), and dietary modifications can be rejected by patients (McCurtin et al., 2018; Wright et al., 2005). In a small comparative study exploring the nutritional intake of 55 patients on elderly and neurology wards in a hospital in England, none of the 30 patients on texture‐modified diets met their energy needs compared with nearly half the 25 patients on normal diets (Wright et al., 2005). Only four of those on texture‐modified diets finished their meals. With respect to modifying fluids with thickening agents, almost all stroke survivors interviewed in another study found this experience to be negative, often in extreme terms (McCurtin et al., 2018).

Existing research provides very little understanding for the mechanisms through which SLTs and nurses interact to share their knowledge of the various risks associated with ongoing dysphagia management. Small‐scale studies at the interface between the professions indicate that enacting swallowing recommendations carries some burden for nurses. A survey about adherence to recommendations, completed by 77 nurses in five hospitals in the United States, reported time constraints associated with supporting patients at mealtimes. The 42% of nurses reporting frustration identified the key reason as time spent supporting patients to eat, with other factors relating to staffing, catering, and reluctance by patients (McCullough et al., 2007). Nurses also experience challenges associated with balancing support for eating and drinking with other priorities, such as medication rounds (Ross et al., 2011). A previous ethnographic study that investigated risk‐related decision making on four medical wards in the UK, suggested that the demands of the ward shaped how nurses reasoned what constituted risk and how to respond to it. The research found that nurses have many potential risks to consider whilst caring for patients within resource‐constrained contexts, leading them to draw on norms and values when making patient safety decisions (Dixon‐Woods et al., 2009).

Normalized practices may come under scrutiny when things go wrong. When people receiving care in health and social care settings are found to be eating and drinking contrary to advice for safe swallowing, clinical staff are urged to complete critical incident reports (Aged Care Quality and Safety Commission, 2021; Care Quality Commission, 2018). The understanding behind encouragement to report actual as well as near miss incidents is the belief that analysing the root causes of errors can provide systems‐level appreciation of issues affecting patient safety. The principles underpinning this understanding have been imported into healthcare from the safety practices of industries such as nuclear power and aviation (Cooke, 2009). ‘High reliability’ principles include viewing safety as central, incorporating redundancies (such as double checks) into safety systems, dispersing authority for responding to risk and learning from mistakes. However, there has been criticism that high reliability principles inadequately account for complexities in the cultural context of healthcare (Liberati et al., 2018). Regulatory controls leave little space for consideration for how risk is socially constructed in context (McDonald et al., 2005). Collaboration may be undermined and interprofessional tension revealed when autonomy is contested (Ewashen et al., 2013), and over‐reliance on formal reporting can create tensions and threaten relationships (Martin et al., 2018). The authors are not aware of previous research exploring in‐depth how SLTs and nurses work together to ensure ongoing safety with eating and drinking for patients with stroke‐associated dysphagia, or the use of incident reporting as a tool for managing deviations from swallowing recommendations.

The question for this research was ‘how is information for ongoing management of swallow safety shared by SLTs and nurses on stroke units across time?’ The objectives were (1) to understand how verbal and written information for swallow safety was shared across time and space, (2) to explore how SLTs and nurses acted when information contained in recommendations was not followed and (3) to understand SLT and nursing perceptions of roles and interdependencies with respect to enacting swallow safety.

## METHODS

### Design

Ethnography was selected as an appropriate methodology due to its focus on understanding the taken for granted ways in which people operate in the context of their everyday lives. Ethnography is increasingly used in applied healthcare settings; it involves the researcher embedding themselves in a setting and collecting different kinds of data (Hammersley & Atkinson, 2019). The analysis was informed by social constructionism; the belief that knowledge and reality are socially constructed (Crotty, 1998). Fieldwork (357 h) was conducted on three stroke wards for 40 weeks between 2015 and 2017. Reporting adheres to the Standards for Reporting Qualitative Research guidelines (O'Brien et al., 2014). An earlier publication reporting a different aspect of the findings includes more expansive discussion of methods (Barnard et al., 2021) (see the additional supporting information).

### Site selection and participant sample

Selection of stroke wards was guided by the aim to include different types of acute settings. Three wards across two inner city National Health Service (NHS) Trusts in England were included (Table 1). Ward names have been changed. Keats admitted patients at stroke onset for around a week, and Shelley and Brooke provided continuing inpatient stroke rehabilitation. SLTs worked Monday to Friday. On Brooke, one SLT and one SLTA covered a 4‐hour Saturday shift, on rotation.

**TABLE 1:** Details of fieldwork and ward settings

Ward	Type of ward	Frequency of interprofessional meetings (Monday–Friday)	Interprofessional patient record
Keats NHS Trust 1; 18 stroke beds; fieldwork: 110 h over 12 weeks	Hyper‐acute: dedicated stroke unit	Daily MDM^b^ and daily brief afternoon meeting to catch up on patients	Paper based
Shelley NHS Trust 1; 17 stroke beds; fieldwork: 124.5 h over 16 weeks	Acute rehabilitation: dedicated stroke bays across two adjacent single‐sex neurology wards^a^	Weekly MDM	Paper based
Brooke NHS Trust 2; 24 stroke beds; fieldwork: 122.5 h over 12 weeks	Acute rehabilitation: dedicated stroke unit	Weekly MDM and daily brief morning meeting 4 days a week to catch up on patients	Electronic

*Notes*: ^a^Nurses located on a single ward (stroke and other neurology). Stroke‐dedicated therapists and doctors.

^b^MDM, multidisciplinary meeting.

SLTs, registered nurses, SLT assistants (SLTAs) and nursing assistants (NAs) were invited to participate in observations and interviews (Table 2). Students were excluded. All 16 SLT staff allocated to or covering the wards during fieldwork over the study period were observed. One SLT left the Trust, thus interviews were conducted with 15 SLT staff. Sampling of nursing staff for observation was dependent on the presence of those on a particular shift who had consented to participate in the study. Participants observed included nurses (50), NAs (7), SLTs (15) and SLTAs (1). One nurse and one NA declined participation. For interview, nursing staff that had been observed were purposively sampled by gender and nursing band (grade) to achieve a diverse sample. Interviews were conducted with 24 nurses and four NAs. Due to shift working patterns nursing staff were recruited throughout the fieldwork periods, whereas most SLTs were recruited at the start. Biographical information has been aggregated to protect identities (Table 2).

**TABLE 2:** Participant information

	Nature of participation			
Participants	Interviewed	Observed	Years of experience^a^	NHS pay band (grade)
16 SLT staff (all female)	14 SLT	15 SLT	Mean = 7.7 years	Bands 7–8 (*n* = 9)
	1 SLTA	1 SLTA	Median = 5 years	Band 6 (*n* = 4)
			Range = 1.5–27 years	Band 5 (*n* = 2)
				Bands 2–3 (*n* = 1)
57 nursing staff (41 female, 16 male)	24 Nurse	50 Nurse	Mean = 8.6 years	Band 7 (*n* = 5)
	4 NA	7 NA	Median = 5 years	Band 6 (*n* = 10)
			Range = 4 months–40 years	Band 5 (*n* = 35)
				Bands 2–3 (*n* = 7)

*Notes*: SLT, speech and language therapist; SLTA, SLT assistant; NA, nursing assistant.

^a^ Information collected for interview participants only.

^b^Registered SLTs and nurses are band 5 and above.

Written patient consent was gained to review SLT and nursing entries in the patient record. Patients were purposively sampled to include a range of severities of swallowing and communication difficulties. The sample included 19 patients, nine men and 10 women. Of these, 14 had swallowing difficulties of differing severities: mild (three), moderate/severe (six), and severe (five).

### Ethical considerations

The National Research Ethics Service and the two NHS Trusts hosting the study provided ethical approval. Information was presented to SLTs and senior nurses in meetings prior to commencing the study and again at the start of fieldwork. Posters were displayed in staff areas and on the wards. Patients were provided with a single page overview of the study to inform them of the purpose of the researcher's presence on the wards. Potential SLT, nursing and patient participants were given information sheets, which were talked through prior to seeking written consent. Data were anonymised at the time field notes were taken and when transcribing interviews.

### Data collection

All data were collected by the first author; referred to henceforth in the first person, as is customary in ethnographic reporting (Clifford, 1986). My position as a SLT–researcher aiming to equitably capture the experiences of SLTs and nurses influenced the role adopted in the field. I participated socially and assisted non‐clinically where I could, for example by picking up the phone when there was no one at the nursing station. However, to reduce over‐alliance with either profession, I remained somewhat marginal to both disciplinary groups (Hammersley & Atkinson, 2019). I had previously been employed as a SLT in both participating NHS Trusts, but not on the three wards and had been a colleague of two of the SLTs. I used a reflexive diary to continually interrogate the lenses through which I observed my own profession and the nursing profession and reflected on insider/outsider positions as they shifted throughout the study (Wind, 2008). This included reflections on how my presence in the field may have influenced how participants behaved or responded to interview questions. Data comprised fieldnotes collected during observation, including hand‐written notes taken from entries in the patient record, and verbatim transcripts of semi‐structured interviews. These data were collected in an iterative manner, such that the direction of inquiry was influenced by emerging insights.

#### Fieldnotes

Fieldnotes captured interactions relating to the common clinical interests of SLTs and nurses that occurred informally and through structured routes, such as meetings and the patient record, and between nurses at nursing handover. I aimed to capture SLT–nursing interactions as they arose, remaining outside of patient areas to protect patient privacy. Fieldnote entries included a combination of captured dialogue and broader observations relevant to understanding the context within which information exchange occurred. Information copied from the patient record formed part of the fieldnote data and included SLT and nurse entries reflecting their common clinical interests.

Most fieldwork periods were of 3–4 h duration (range = 1–12 h), Monday to Friday. I often started with the nursing staff, at 0715, as handover assisted field relationships. Preliminary analysis 2 months into the first fieldwork period indicated that more understanding was needed for the context in which swallowing information was used and fed forward on days when SLTs were not at work. Thus, observations took place across the 7‐day week. General observations occurred wherever SLTs and nurses operated, such as nursing stations and meeting rooms. More directed observations involved shadowing SLTs as they moved about the ward and short periods observing specific nurses to better understand the context of their work. Handwritten fieldnotes were typed out in full at the end of each day.

#### Interviews

Semi‐structured interviews were completed with 14 SLTs, one SLTA, 24 nurses and four NAs. A broad topic guide provided a flexible structure around which evolving areas of interest could be explored. All participants were asked questions relating to their information‐sharing practices, roles, relationships, and interests in common. However, in accordance with the iterative ethnographic approach, specific lines of enquiry developed over time. Interviews were audio‐recorded and transcribed by the first author. They were 21–55 min in length.

### Data analysis

Data were analysed using techniques from the constant comparative method in which data similarities and differences were considered within and across data sources (Hammersley & Atkinson, 2019). Fieldnotes and interview transcripts were read repeatedly. With the support of qualitative data management software, analysis commenced with open coding, before applying more focused coding and developing categories. Categories were iteratively revised as additional data were collected (Hammersley & Atkinson, 2019). Analysis switched to a paper‐based process at the end of fieldwork in which patterns, relationships, and contradictions were explored (Hammersley & Atkinson, 2019; Thorne, 2016). Notes taken from the patient record were colour coded by hand and interrogated in association with other data during the paper‐based stage described above. Data were organized into themes, illustrated through interview quotes and fieldnote extracts. Due to the risk that in‐depth reporting could reveal identities, biographical information attached to quotes is restricted to a gendered pseudonym and years of experience. Where interview extracts have been truncated for brevity, this is indicated by ‘(…)’. Observational data from field notes are identified as [Fieldnote: date recorded].

### Rigour

Credibility of interpretations was enhanced through multiple approaches (Hammersley & Atkinson, 2019). These included prolonged engagement, triangulation of different sources of data, active search for and examination of negative cases, keeping a reflexive diary, discussion of preliminary findings with participants at interim periods, and ongoing discussion with the research team. The potential for findings to be transferred was increased through rich description and inclusion of multiple sites.

## FINDINGS

An explanation was created for the influence of disciplinary differences in time and space on how SLT and nursing staff managed their shared interest in dysphagia‐associated risks. Findings are organized around three themes: (1) SLTs and nurses were aligned in concern for swallow safety across all information‐sharing routes; however, (2) ambiguity was introduced by the need for information contained in swallowing recommendations to travel across time, with consequences for (3) critical incident reporting and relationships. Staff are henceforth referred to as SLTs or nurses, except where assistants are specifically referenced.

### Alignment in concern for swallow safety across all information‐sharing routes

The information SLTs and nurses shared about patients’ swallowing was valued by both disciplines. It was needed to safely execute tasks relating to mealtimes, hydration, and medication. During interviews, explanations of their own and each other's roles with swallowing concurred. Both disciplines viewed SLT roles as assessing, advising, and reviewing. Both viewed nursing roles as implementing recommendations, monitoring how patients were managing, liaising with family, and flagging concerns. Nurses on Keats also conducted swallow screening. Each discipline was very aware of the risks of aspiration or choking and respect for each other's roles was evident.

I think we give extreme importance (to the relationship with SLT) because of the airway and eating and choking. [Grace, 10 years, nurse]

I would feel I have confidence in the stroke nursing staff to be able to detect changes in a patient's clinical status secondary to a dysphagia or aspiration pneumonia. [Leanne, 10 years, SLT]

On all wards, both disciplines made use of all available verbal and written routes for swallowing information. See Table 1 for differences across wards with respect to meetings and the patient record. They both considered it important to share information about swallowing. They commonly sought each other out to update on patient status and brought information to meetings. Nurses often mentioned swallowing in nursing handover and referred to it in the patient record. Such information might, for example, include recommended consistencies or signal when SLT input was needed. On Keats, information frequently related to swallow screening. SLTs almost always reported both assessment and advisory information in the patient record. They summarized recommendations on bedside signs, which nurses highly valued and usually trusted. When recommending changes, SLTs usually also conveyed information verbally, and with high‐risk decisions, they emphasized the need for caution through several information‐sharing routes.

I'll make sure that it's verbal as well as written and it's on the CDR (clinical data repository), and it's communicated in as many ways as possible. [Tamsin, 5 years, SLT]

### The need for information to travel across time introduces ambiguity

The making and enactment of swallowing recommendations were separated in time. SLTs were often not present when nurses attempted to offer food, drink, or medication in the advised manner and patient‐facing realities placed demands on nursing staff.

#### Competing demands

SLTs made recommendations based on assessment of individual patients. Recommendations commonly included food and fluid modifications, procedural advice for how to eat and drink (e.g., upright position, or prompt to swallow), indicators of risk (e.g., cough or shortness of breath) and the action to take should patients show signs of not coping (e.g., place nil by mouth). Nurses considered swallowing recommendations to be important and tried to make time to hear or read them and ensure they were adhered to. However, they owed a duty of care to *all* the patients in the bay they were managing. They usually ensured patients had the right consistencies of food and drink but faced routine difficulties in maintaining optimal conditions. They often supported more than one patient at a time, increasing the potential for harm for patients who had capacity to feed themselves but were recommended to receive close supervision. In such circumstances, nursing staff demonstrated awareness for safety by calling across reminders to patients or making a delayed intervention.

One of the NAs on Keats was alternating between close and distant supervision for a patient who had a bedside sign recommending he eat slowly, under strict supervision. Whilst the NA was busy with another patient and the nurse was occupied at the nursing station, the patient coughed three times. No one seemed to register the first cough; the second time the NA looked towards the patient, and on the third cough the nurse went over and reminded him to eat slowly [Fieldnote: 121016].

The need to move between patients reduced the ability of the NA to respond to the cough, the most overt sign that the patient was not coping. His reduced responsiveness did not appear to reflect lack of knowledge or competence. I had observed him earlier carefully explaining to the hostess (the person who hands food and drink to patients) why she needed to check the sign on the wall before offering tea to patients on the stroke ward. On another occasion, he asked the SLT to change a bedside sign because it represented a risk, being written in light ink and hard to read. His actions indicated real‐time difficulties in managing the mealtimes of several patients.

SLTs appreciated that their recommendations were just one of the many demands on nurses. During interview, they empathized with nurses’ competing priorities. Nonetheless they were obliged by their own duty of care to recommend the safest way for patients to eat and drink and needed to have trust in nurses’ vigilance. They encouraged a cautious approach when risks were high:

Because it's on my head, like I'm the one who's balanced the risk and decided what they're to have, but (…) they're the people who are carrying it out (…). If someone's a bit borderline, I'll be like, ‘please be very careful, if there's any problems just put them nil by mouth’. [Mary, 6 years, SLT]

#### Patients improve and deteriorate

Patients could improve or deteriorate in the time since SLT recommendations were made. Nurses needed to make decisions in that moment, yet SLTs were present intermittently. There were constraints on how nurses could legitimately act because the recommendation carried a certain authority, representing the most recent specialist assessment. SLTs usually left some advice for what to do if patients did not cope well with their recommendation, but this did not help when risks were not clear cut. There was little direction for what to do if patients improved.

Over weekends or public holidays, it could be several days before SLTs returned to the ward, creating clinical dilemmas for nurses. One of the nurses explained that when faced with a patient who was not coping with recommendations over a weekend, she would confer with her nursing colleagues, and if the risk was clear, seek a medical review. However, she might make her own clinical judgment if ambivalent.

Policy says that we should keep them nil by mouth if we're worried about their swallow (…), but if this is a Saturday morning, we don't really want them nil by mouth for the whole time (…), in practice I've found that we've given medication to them in the easiest possible way, so we've given it to them with thickened fluids, and we've crushed the medication if we're at all worried about it, but the minimum amount that's possible, so it's also minimising the risk to the patient, and they'd be on IV fluids or whatever until they were actually able to be assessed. [Amaya, 2 years, nurse]

When asked what she would hand over to the next shift, she suggested that she would allow the oncoming nurse to make their own professional judgment about whether to give the medication with a thicker consistency or keep the patient nil by mouth. Thus, she did not consider her judgment to be authoritative, and uncertainty in managing the patient might travel through each nurse until the SLT was back and able to reassess.

### Critical incident reporting and relationships

SLTs commonly reported coming to review patients and finding they were not adhering to the recommended advice. They experienced this across the hospital, as well as on the stroke wards, and it created uncertainty for how to act. When SLTs felt that nurses had not been sufficiently vigilant, the need for a difficult conversation could create unease in the SLT–nurse relationship.

I go and see her (the patient), and she's literally got a massive sausage in her mouth, and she can't chew. She's been there coughing and choking, and she was supposed to be nil by mouth waiting for speech therapy, and I sort of, I said to the nurses, well why has, I was a bit like, what's going on (…) so the question is how do I approach it? [Irene, 13 years, SLT]

Both disciplines reported challenges associated with occasions when patients urged nurses to upgrade their eating and drinking regime. When SLTs were not available, nurses needed to balance their clinical evaluation of risk with patients’ wishes and they felt compelled to find a way to manage the situation as they encountered it. However, using their own clinical reasoning risked negative consequences, as their actions could result in harm or be considered a near miss critical incident. During fieldwork, one of the SLTs narrated an incident that I had the opportunity to explore when interviewing the parties involved. The SLT explained how she had recommended oral trials of only five teaspoons at each meal for a patient admitted on a Friday, due to a fatigue effect on the swallow. Oral trials are tightly controlled trials of food or fluids for patients who are not otherwise ready for oral intake. SLTs cautiously accept a higher level of risk for these patients for the purpose of keeping the swallow mechanism active. When the SLT sought an update from the nurse on Monday, the nurse advised her that the patient had been eating over half of her meal. This led to an encounter the SLT found awkward. She explained to the nurse that due to the potential for harm, she needed to report it as a critical incident.

During interview, the senior nurse responsible for investigating the incident explained that the patient had been asking to eat more of the meal, placing the nurses in an uncomfortable position between the desires and agency of the patient and the swallowing recommendation. The nurses at the weekend had made a clinical judgment that the patient could tolerate more than recommended because she did not cough or appear tired when eating. From the SLT perspective, it was concern for fatigue in swallow function (rather than tiredness) that had caused her to make a conservative recommendation for trials of very small quantities. The investigating nurse explained the nurses’ dilemma with respect to their patient‐facing realities:

The nurses they didn't know what to do. In the end they fed the patient as she wishes to, but of course when the speech language therapist came, they say ‘no you shouldn't do that, you shouldn't do that’ (…) because they feel that the patient will be weak, will be this and this (…) so it becomes a big issue, a big issue, ‘oh, you've fed the patient too much’, but she's asking for it and she's not coughing, and we can't see any problem. [Ruth, 10 years, nurse]

The tension between SLT expectations that nurses keep to the recommended advice, and space for clinical reasoning by nurses, is evident in what she goes on to say:

The speech language therapist puts instruction there, you have to follow them, no matter what the patient wants, you know, so even if it's an uncomfortable situation you have to stick to that (…). They feel that if we've been given instructions and you don't follow, or something happens (…) you will cause problems for everybody. [Ruth, 10 years, nurse]

The nurse alludes to the spectre of reporting as a pervasive threat and conveys a dilemma between acting in patient‐centred ways and avoiding negative consequences. SLTs found raising incidents uncomfortable. They experienced dilemmas between faith in the regulatory process and concerns that raising incidents could be detrimental to their relationship with nursing colleagues. The following extracts illustrate differing SLT beliefs in reporting as an influencing tool:

I think still having a verbal conversation would be better, rather than filing something on a computer system that goes off to whoever. [Tamsin, 5 years, SLT]

If there is a problem and that problem's being highlighted and raised enough times and it comes back ‘well we were short staffed this day, this day, this day’, then that means that there's more staffing on the ward and more cover, then that's gonna be great for everyone. [Rhea, 3 years, SLT]

SLTs differed on an individual level in their approaches to reporting, sometimes deciding not to file a report to preserve a relationship with nurses that could be seen as fragile. The most experienced SLT in the study was the least conflicted in her handling of incidents. She explained her preference for a more local response for resolving issues where possible:

I say to the staff (…) ‘think about what you want to do. Do you want to do a (incident report) which will take this matter out of your hands, or do you want to resolve this right here and now, with this nurse?’ Sometimes you can do both, but sometimes the problem with (incident reports) is that they are often seen as punitive, and where there is a need to restore something, to teach something, to regain some skill and confidence, there's maybe a better way. [Pam, 27 years, SLT]

Although both disciplines understood at an intellectual level that reports were directed at incidents and both reported incidents of potential harm, reporting also felt personal. SLTs experienced a dilemma between taking action that could potentially lead to improvements in patient safety and maintaining a relationship with nurses.

## DISCUSSION

This study has provided new understanding for dilemmas associated with temporal gaps between provision of swallow safety information through recommendations, enactment of recommendations, and subsequent interactions. Nursing dilemmas related to enacting recommendations and autonomy for in‐the‐moment decision‐making in response to patient‐facing realities. SLT dilemmas related to actions to take if recommendations were not followed. Swallowing management was influenced by reduced coverage of SLTs across the week and their distance from routine eating and drinking by patients. Information contained in recommendations was frozen in time until SLTs reassessed, and competing demands at mealtimes challenged consistency of optimal conditions. This discussion will draw on narratives of patient safety and principles from bioethics to explore these dilemmas further.

The research has demonstrated that both disciplines acted in accordance with the belief that swallow safety could be enhanced through high reliability safety principles. They showed concern for the risks associated with swallowing, they shared information through a range of routes to add redundancy, and they made use of the incident reporting process (Cooke, 2009). Each discipline viewed their duty of care towards the risks from swallowing in terms of setting and keeping to guidelines, in line with *deontological* ethical principles, wherein value is attached to rule‐directed behaviour (Beauchamp et al., 2014). However, swallowing management was characterized by uncertainties that could often not be resolved through following prescribed recommendations (or *rules*) derived from assessment in best‐possible conditions at a particular point in time. Ambiguity was a routine feature of swallowing management because mealtimes required nurses to manage competing demands and patients improved or deteriorated over time.

Previous ethnographic research conducted in hospital settings indicates that when making decisions about the many potential risks associated with patient‐centred care, nurses sometimes consider ‘tolerating some trouble’ as necessary for getting things done, in a context of resource constraints (Dixon‐Woods et al., 2009:367). When there are insufficient staff to meet the needs of patients, moral principles can conflict, creating ethical dilemmas (Beauchamp et al., 2014). In the current study, nurses acted with an obligation to allocate time and attention to all patients in their care, not just those who needed mealtime supervision, potentially pitting the ethical principle of *justice* (fair distribution of resources) against the principle of *beneficence* (doing good) for individual patients. In contrast, SLTs made recommendations for individual patients referred to them. They did not need to consider the potential impact of additional time supporting that patient on time available for the other patients.

Nurses’ roles in coordinating and implementing information provided by the numerous healthcare professionals they interact with often remain hidden to those professionals (Allen, 2014). The current study revealed a complex relationship between SLT‐led edicts intended to increase swallow safety and the less visible pragmatics of care provision. Consistent with current debates in the literature, swallowing recommendations have potential to complicate medical care, for example, the need to give medication or optimize nutrition (Atkinson & O'Kane, 2018; O'Keefe, 2018). In addition, when patients pressed their desire to eat and drink in ways that conflicted with SLT advice, this created disruption between nurses’ obligations towards swallowing recommendations and to patients as individuals.

Previous research has revealed how on‐the‐ground realities impact on nurses’ capacity to deliver safe care (Dixon‐Woods et al., 2009) and their need to be responsive to patients may morally compel them to deviate from role boundaries or plans of care made by others (Barlow et al., 2018; Peter & Liaschenko, 2004). This may lead to actions that could introduce risk. For example, the addition of thickening agents is commonly seen as the go‐to treatment for swallowing difficulties (O'Keefe, 2018). SLTs are currently questioning overreliance on this practice and encouraging fuller use of other management options (Atkinson & O'Kane, 2018). However, the frequency with which SLTs recommend thickened fluids may lead nurses to erroneously view this as the safest option when faced with an in‐the‐moment decision. If nurses’ ethical dilemmas remain hidden, other professionals will continue to be unaware of moral quandaries associated with their proximal position (Peter & Liaschenko, 2004). Cross‐disciplinary discussion about pragmatic realities could help increase adherence to swallowing advice and thus reduce risks of aspiration or choking.

Ambiguity and uncertainty created issues for nurses in real time that they needed to resolve to effectively perform their clinical roles and preserve the integrity of the nurse–patient relationship. Understanding patient safety through a relational lens was promoted in the ‘To Care is Human’ report (Wolf, 2018). The report deliberately played on the title of an earlier highly influential report directing healthcare towards safety improvements through reporting and learning from errors, ‘To Err is Human’ (Kohn et al., 2000). ‘To Care is Human’ emphasized that patients want to be listened to at the same time as having confidence that those treating them can meet their healthcare needs and protect them from harm (Wolf, 2018). This is consistent with increasing attention towards helping patients make informed choices about recommendations intended to reduce risks from swallowing (Speech Pathology Australia, 2019). However, meeting nursing professional standards for keeping patients safe from harm at the same time as responding to them as individuals with preferences (Nursing & Midwifery Council, 2018) may be more challenging in the early acute stages of stroke care and when there are questions about patients’ mental capacity for swallow‐related decisions.

SLT dilemmas differed from those of nurses. They made recommendations based on judgments of risk following assessment and felt bound by professional standards for establishing safe care to take follow up action of some kind when recommendations were not followed (HCPC, 2014). However, incidents usually related to the potential for, rather than actual, harm and SLTs needed to consider whether to let things go, resolve through conversation, or complete an incident report. During interviews SLTs indicated that encountering patients eating or drinking contrary to recommendations could create strain on relationships with nurses. Tensions were not usually overt. When determining what action to take, SLTs attempted to strike a balance between encouraging vigilance and preserving the interprofessional relationship. Despite best efforts to disassociate clinical error from blame, feeling culpable when things go wrong is a human reaction that is hard to avoid. Incident reporting can carry an emotional toll and uncertain consequences for both reporter and reportee (Dixon‐Woods et al., 2009; Martin et al., 2018). SLTs were aware that as the creators of swallowing recommendations, they held a certain authority; however, they often felt uneasy about this, and its impact on relationships.

The nurses in this study did not make decisions to deviate from recommendations lightly. They were aware that acting autonomously carried potential risks to patients and to themselves should an incident be raised and took account of different factors when determining the acceptability of routinely encountered risks. These factors might include experience, training, time, or staffing (Arfanis et al., 2011). When SLTs were absent, nurses were reliant on resources available to them within that shift, in a context which limited their autonomy for acting in‐the‐moment in ways they considered most beneficial to patients. Research exploring differing risk perspectives across professional groups has indicated that ‘some discourses are afforded more legitimacy than others’ (Rowland & Kitto, 2014: 332). In the current study, the information contained within recommendations dominated discourses surrounding swallow risk, with critical incident reporting a sanctioned route for learning from error. Organizational oversight of errors through reporting systems is important. Learning has potential to benefit future patients and may encourage professionals to remain vigilant to risk (Hewitt & Chreim, 2015). However, regulatory processes may not in fact result in safety improvements, and reporting can have unintended relational consequences, particularly when no harm has arisen (Martin et al, 2018). Excessive reporting of incidents of potential harm may have less benefit to practice improvement than localized discussions about how risk is interpreted in‐context (McDonald et al., 2005). The most meaningful risk controls may not be the regulatory controls that top the patient safety hierarchy, but *softer* signals that lay at the bottom, for example improving safety through skill mix or teamwork (Liberati et al., 2018). Previous in‐depth exploration of safety signals within regulatory practice indicates that encouraging ‘collective sensemaking’ through team discussion of soft signals could provide important information about the ambiguities and relational issues that contribute to risk complexities (Kok et al., 2020). When soft intelligence is gathered locally for the purpose of understanding the complexities of risk, it can create trust and improve processes in ways that are harder to achieve through reporting alone (Martin et al., 2018).

Potential systems‐level changes could include advanced competencies for nursing staff to act in the absence of SLTs (Boaden et al., n.d.), increasing capacity at mealtimes through increased staffing or use of volunteers (Edwards et al., 2017), or increasing SLT presence across the 7‐day week (Gittins et al., 2020) and at mealtimes. However, these are not simple solutions; covering 7 days without additional staffing, would result in thinner SLT cover across the week (Gittins et al., 2020), and extending nursing roles can be both empowering and a burden, as seen in nurse prescribing (Dowden, 2016). In the immediate, changes could arise from creating space for SLTs and nurses to discuss the dilemmas experienced by the other and explore local solutions. SLTs could better understand nursing realities by moving closer to nursing space, either physically or through hearing their experiences (Peter & Liaschenko, 2004) and mutual learning could increase nurses’ appreciation for SLTs’ relational concerns.

### Limitations

A particular strength of the ethnographic methodology was that the relationship between observed and reported behaviour could be explored. In addition, a concerted effort was made to identify both corroborative and contradictory evidence during fieldwork and interviews. The units of analysis in this study were SLTs and nurses rather than ward comparisons. We actively searched for contradictory data to ensure findings did not hide substantive differences in information‐sharing behaviours across the three wards. However, understanding of a different kind would have been created had the wards been the units of analysis. Findings are contextually situated. This account is partial for reasons which include the following: the nursing sample represented just a proportion of the nursing complement; not all interactions over the fieldwork period will have been captured; interpretations were filtered through a disciplinary lens (Thorne, 2016), and there may be systems level influences that were not explored, for example, all wards were in teaching hospitals in urban areas. A criterion for considering the transferability of ethnographic work is the extent to which explanations of phenomena are plausible (Greenhalgh & Swinglehurst, 2011). In this study, rigorous attention to enhancing credibility and the application of principles from bioethics and narratives of patient safety to the findings has provided a plausible explanation of the dilemmas experienced by SLTs and stroke nurses. Preliminary findings resonated with staff on the studied wards and can be expected to resonate with staff on other stroke wards, and potentially in acute settings more broadly. Finally, the authors are not aware of research published since data collection ended in 2017 that would question the plausibility of these insights into how SLTs and nurses on stroke wards work together in the ongoing management of dysphagia on stroke wards.

## CONCLUSIONS

The SLTs and nurses in this study understood the risks associated with swallowing and patient safety was important to them. However, disciplinary differences in temporal–spatial experience and responsibility for swallowing recommendations influenced their conceptions of risk and options for managing it. SLTs were distanced from the consequences of their recommendations, and the on‐the‐ground dilemmas of nurses were often hidden. The application of debates within the patient safety literature between hard and soft patient safety signals to the risks from swallowing provides a foundation for collaborative discussion about ethical dilemmas, the nature of risk and interprofessional relationships. Such discussion could help teams explore how information for swallow safety could be kept up to date and implementable within ward realities. Possibilities include considering nursing capacity to act when SLTs are not there, mealtime staffing, and SLT 7‐day working. Together they can review their understanding of risk and preferred local and formal routes for learning from it. Future survey‐based research could establish the extent to which issues raised by this study apply more broadly. Additionally, focus group research could help identify potential solutions. Future studies would benefit from including patients’ perspectives on swallow risk and exploring patient involvement in the making and enactment of recommendations.

## CONFLICTS OF INTEREST

No potential conflict of interest has been declared by the authors.

## FUNDING

This study was funded by a City, University of London Doctoral Studentship. This research received no specific grant from any funding agency in the public, commercial or not‐for‐profit sectors

## Supporting information

Supporting Information

## Data Availability

Research data are not shared.
