# Supplementary material for: Managing ongoing swallow safety through information‐sharing: An ethnography of speech and language therapists and nurses at work on stroke units
Source: Int J Lang Commun Disord. 2022 Apr 9;57(4):852–64. doi: 10.1111/1460-6984.12725 (PMC9541144; doi:10.1111/1460-6984.12725)
Supplement: Supplementary file 1 — Supporting Information [file JLCD-57-852-s001.docx]

Supplement 1 Standards for reporting qualitative research (SRQR)

O'Brien, B.C., Harris, I.B., Beckman, T.J. & Reed, D.A. (2014). Standards for reporting qualitative research: A synthesis of recommendations. *Academic Medicine, 89*(9), 1245-51.

| Checklist item | Location |
| --- | --- |
| S1 Title  Concise study description, identified as ethnography | Title |
| S2 Abstract  Key study elements included | Abstract |
| S3 Problem formulation  Description and significance described, with empirical and theoretical support | Introduction Background |
| S4 Purpose  Objectives, research question and aims included | Background |
| S5 Ethnographic methodology and social constructionism briefly explained | Methods |
| S6 Researcher characteristics and reflexivity  Researcher profession and relationship to the field and use of reflexive diary briefly explained. | Methods Limitations |
| S7 The context is described - the three wards | Methods  Table 1 |
| S8 Sampling strategy  Explanation of how nurses and SLTs sampled for observation and interview and sampling patients with respect to access to their patient record. | Methods |
| S9 Ethical issues pertaining to human subjects  Information provided re. regulatory approvals, explanations of processes for introducing the study and gaining consent and protecting identity | Methods |
| S10 Data collection methods  Described the different types of data collected and the iterative process as the study evolved. | Methods |
| S11 Described iterative approach to use of topic guide. | Methods |
| S12 Information about participants provided in composite to protect identity | Table 2  Methods |
| S13 Explained data processing, management of fieldnote and interview data. | Methods |
| S14 Described how constant comparative method used to analyse data to generate a thematically organised explanation. | Methods |
| S15 Techniques to enhance trustworthiness explained | Methods |
| S16 Interpretations clearly presented throughout main findings | Findings |
| S17 Quotations and extracts to illustrate interpretations are clearly identified. | Findings |
| S18 Main findings summarised, and explanation provided for how they extend previous work and create new understanding for how SLTs and nurses manage swallowing with suggestions for clinical practice. | Findings |
| S19 Trustworthiness and limitations of findings | Limitations  Method |
| S20 Conflicts of interest statement | Statement |
| S21 There is no external grant funding to declare for this study (doctoral research funded by studentship) | Declaration on submission to journal |
